# I Trust You: Does This Matter in the Relationship between Sexual Harassment, Continuous Commitment and Intention to Leave among Young Female Healthcare Professionals?

**DOI:** 10.3390/ijerph19052843

**Published:** 2022-03-01

**Authors:** Hassane Gharbi, Nadir Aliane, Abu Elnasr E. Sobaih

**Affiliations:** 1 Management Department, College of Business Administration, King Faisal University, Al-Ahsaa 31982, Saudi Arabia; hgharbi@kfu.edu.sa (H.G.); nhaliane@kfu.edu.sa (N.A.); 2 School of Business, University of Sfax, Sfax 3018, Tunisia; 3 Faculty of Tourism and Hotel Management, Helwan University, Cairo 12612, Egypt

**Keywords:** healthcare, social exchange theory (SET), sexual harassment, trust in superiors, continuous commitment, intention to leave

## Abstract

This research examines the direct influence of sexual harassment by superiors on subordinates’ young female trust in their superiors. The research also examines the mediating role of trust in the relationship between sexual harassment and continuous commitment as well as intention to leave. For this purpose, a pre-tested questionnaire survey was self-dropped and collected by the research team to young female professionals, who are in their early career (within five years of their career), in public hospitals in the cities of Tunis, Sfax and Sousse, Tunisia. The results were analyzed using SPSS and AMOS. The results of structural model, interestingly, showed no significant effect of sexual harassment by superiors on their subordinates’ trust. Hence, trust in superiors has no mediating role in the relationship between sexual harassment and continuous commitment as well as intention to leave. However, sexual harassment by superiors was found to directly and positively influence young female professionals’ intention to leave the job. Additionally, trust in superiors was found to negatively influence both young female professionals’ continuous commitment and their intention to leave. The results have certain theoretical and managerial implications, particularly in relation to young female professional in the healthcare sector, which is vital for Tunisia and every country.

## 1. Introduction

Healthcare is a unique industry, whose success depends on competent employees. Recent statistics [1] showed that women make most of the healthcare employees globally, especially at the entry level, but the proportion varies between countries. However, almost half of women experienced sexual harassment behavior at their workplace [2]. Young female health professionals are more likely to experience violence and sexual harassment at their workplace than other health professionals [3]. Research studies (e.g., [4,5]) showed that young and unmarried female are likely to experience sexual harassment in comparison to other females or other employees. Additionally, young female healthcare professionals can be harassed by their patients, colleagues and/or their superiors [6,7]. Thus, research [2] confirmed that they are at the highest volume of sexual harassment among the healthcare professionals. Nonetheless, this unwelcome and offensive behavior could negatively affect attitudes and behaviors of young females [8]. As a result, they could feel uncomfortable in their jobs [9], distrustful of their co-workers and/or superiors [10,11], and less committed to their organization [12]; this ultimately affects their intention to leave the job [2].

This research draws on social exchange theory (SET) and leader–member exchange (LME) to understand and examine the working relationship between subordinate female health professionals and their supervisors, especially young female doctors, who are under-researched in this context, since a plethora of research focuses on nurses [2]. Social exchanges between superiors and their subordinates evolve and sustain the dyadic relationship. This relationship is based on the idea that leaders and/or superiors in healthcare sectors should offer something to their employees, which they see as valuable, and each party sees the exchange as reasonably equitable or fair [13]. Thus, young females are expected to exhibit positive attitudes and behaviors if they perceive proper practice from their superiors. On the other side, if employees perceive inappropriate practices from their superiors, they will respond by negative attitudes and behaviors toward their leader and colleagues [14].

This research examines the influence of sexual harassment by superiors on subordinate female health professionals’ trust in their superiors. More specifically, it examines the mediating role of trust in superior in the relationship between sexual harassment and continuous commitment and the intention to leave. Trust derives from a range of subjective evaluations inherent in one’s relational background through which a person (in this case, a young female health professional) decides whether to trust or not. Yet, sexual harassment by superiors toward subordinate women compromises their complex moral and emotional commitments. In extreme cases, some of them may not choose to adopt a continuous commitment, whereas others could leave their job. Sexually harassed young female health professionals will react by actively looking for working alternatives [2]. 

A review of the literature to date shows some connections between organizational trust and the intention to leave [15] and, on the other hand, between trust and continuous commitment [16]. Notwithstanding, to the best of the researchers’ knowledge, though not exhaustive, there is no published research that combines all these three factors at the same time, where sexual harassment is the explanatory variable. Consequently, the main research questions are as follows: What is the impact of sexual harassment by superiors on trust in superiors, continuous commitment and the intention to leave? Does trust in superiors play a mediating role in the relationship between sexual harassment by superiors and continuous commitment and the intention to leave? 

This research examines the direct impact of sexual harassment by superiors and on young female health professionals’ trust in superiors. Additionally, the research examines the direct impact of trust in superiors on continuous commitment and intention to leave among young female health professionals. Furthermore, the research examines the indirect impact of sexual harassment on continuous commitment and intention to leave through trust in superiors. A proper understanding of these relationships will help in maintaining appropriate relationships between leaders and their employees and create a healthy and psychologically safe working environment for young female health professionals. 

## 2. The Theoretical Framework and Hypotheses Building

### 2.1. Sexual Harassment and Trust in Superiors

The World Health Organization has identified the harasser in the health sector as a perpetrator of harassment, who can be in authority, respected, and trusted by his/her female subordinates [17]. This harasser, who is in a senior position, can be a man or a woman. Research (e.g., [18]) has confirmed that sexual harassment can be a result of exercising personal power by a superior over subordinates. For example, a recent report showed that many nurses reported sexual harassment by their physician superiors [19]. Notwithstanding, many nurses do not, or do not want to, report these unethical and violent behaviors [20]. 

Sexual harassment can affect young female health professionals in a number of ways. This could include mental and physical health as well as advances in their career [2]. They can suffer negative psychological effects, such as depression, headaches and lowered self-esteem [9,21]. One of the major impacts of this sexual harassment is distrust in superiors and co-workers [10,11]. Studies [10,11] confirmed that experiencing sexual harassment by superiors and or co-workers will result in lower level of trust in both superiors and co-workers. Trust in superiors is a key construct that indicates a positive working relationship; however, this could be disturbed by harassing subordinates [10]. Based on these arguments, the following can be argued:

**Hypothesis** **1.** *Sexual harassment by superiors negatively influences young female health professionals’ trust in their superiors.*

### 2.2. Sexual Harassment and Intention to Leave among Young Female Doctors

As highlighted earlier, sexual harassment has a negative consequence on employees’ mental and physical health [21]. According to SET framework, if employees perceive negative practices from their superiors, they are more likely to respond with negative attitudes and behaviors [13]. A recent study [12] showed that if superiors harassed their employees, employees were more likely to respond with negative attitudes toward their organization and were more likely to have turnover intention. Based on this, the following can be hypothesized:

**Hypothesis** **2.** *Sexual harassment by superiors positively influences the intention to leave among young female health professionals.*

### 2.3. Trust in Superiors and Continuous Commitment among Young Female Doctors

A recent study on Korean employees [12] showed that employees harassed by their leaders are less likely to be committed to their organization, especially in their affective commitment. This was confirmed by another recent study [22] that showed that leaders have a significant influence on their workers’ outcomes directly and indirectly through the inclusive work climate that they create. There is evidence that sexual harassment by superiors is correlated with negative physical and nonphysical outcomes among subordinates, including their commitment toward their organization and whether they are going to continue with their organization or not [23]. Research confirmed that if the employees’ trust increases, the level of continuous commitment will decrease [24]. According to Meyer et al. [25], when continuance commitment is high among employees, they are going to stay in the organization because they have no better option outside the organization. However, if a good opportunity exists, they will leave the organization. This means that the decisions of employees to stay within their organizations are because of the financial costs of changing jobs. Continuance commitment is assessed based on the calculation of costs and benefits and, hence, is more likely to correlate negatively with trust from superiors [25]. In other words, trust in superiors negatively affects employee continuous commitment. Based on this argument, the following can be argued:

**Hypothesis** **3.** *Trust in superiors negatively influences continuous commitment among young female health professionals.*

### 2.4. Trust in Superiors and Intention to Leave among Young Female Doctors

Trust (in organizations and superiors) is a predicator of intention to remain/leave the organization [26]. Research has shown that the different types of trust (trust in organization, co-worker trust and trust in superiors) have a significant relationship and influence on turnover intentions [27]. In another study, trust in superiors was found to have a moderating role in the relationship between job demand and the intention to leave [28]. A negative correlation was confirmed between trust in the organization and the intention to leave [29]. Based on these results, the following can be argued:

**Hypothesis** **4.** *Trust in superiors negatively influences young female health professionals’ intention to leave.*

### 2.5. The Role of Trust in Superiors in the Relationship between Sexual Harassment and Continuous Commitment and Intention to Leave

**Figure 1:**
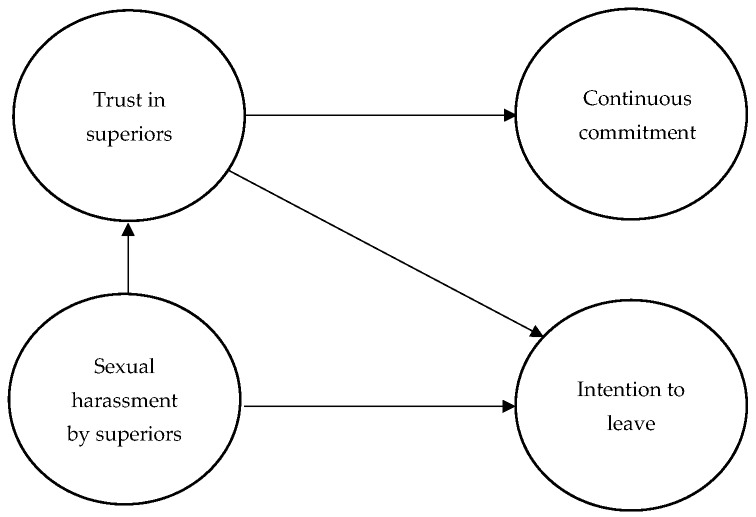
The research conceptual model.

Research has confirmed that sexual harassment by superiors is a predicator of distrust in superiors [10] and the intention to leave [12,21]. On the other side, trust in superior is a predictor of organizational commitment [12,22] and the intention to leave [26,27,28]. Additionally, research also confirmed a relationship between sexual harassment by a superior and the intention to leave [12], but no research, to the researchers’ best knowledge, confirmed a direct influence of sexual harassment by superiors and the continuous commitment of young female professionals. Hence, this research expects that trust in superiors could have a partial mediation effect between sexual harassment and young health female subordinates’ intention to leave the job. On other hand, trust in superiors is expected to have a full mediation effect between sexual harassment and female subordinates’ continuous commitment. These hypotheses are based on the SET framework, which asserts that subordinates are more likely to respond with attitudes and behaviors to the practices undertaken by their superiors (see Figure 1). Thus, the following can be hypothesized:

**Hypothesis** **5a.** *Trust in superiors fully mediates in the relationship between sexual harassment and continuous commitment.*

**Hypothesis** **5b.** *Trust in superiors partially mediates in the relationship between sexual harassment and the intention to leave.*

## 3. Methodology

### 3.1. Data Collection 

The research adopted a survey approach to achieve the purpose of the research. The survey was self-administered by young female doctors who are early in their career in healthcare (often within five years of their career). Five hundred questionnaires were distributed to young females in healthcare. The questionnaire were self-administered by young female health professionals in public hospitals in the cities of Tunis, Sfax and Sousse, Tunisia. In sum, 305 usable questionnaires were collected and deemed valid for analysis, which is a 61% response rate. The survey was distributed and collected by the research team members, who had access to the participants through their personal network. The research team confirmed that the collected data for research purposes and the respondents are anonymous. Hence, no personal data were collected from the respondents. Additionally, to protect the privacy of the hospitals and their participants, all personal information was eliminated from the analysis.

### 3.2. The Research Instrument

The sorting of appropriate measurement scales is difficult. In fact, this stage is particularly important, as it influences the quality of the research. The more that measurement scales are valid and reliable, the more that the information is significant and consistent. In order to conduct empirical quantitative research, the appropriate scales to measure the selected variables were adopted (see Table 1).

### 3.3. Data Analysis

The data collected in this stage of our research were first processed by means of the software SPSS 23. The factorial structure of the various scales was analyzed. In this context, the principal component analysis (PCA) with Varimax rotation was analyzed. The Kaiser criterion was programmed to only consider factors whose value is over “1”, which was accepted to fix the number of factors. As for the internal reliability of the scales, it was calculated using Cronbach’s alpha. 

Furthermore, the data collected were subjected to confirmatory factorial analysis to verify whether the factor structure, which appeared during the explorative phase, was valid. The quality of the scale adjustment was then estimated using structural equation models (SEM) through the software AMOS 23. A series of statistical indicators, widely used in literature, made it possible to evaluate the good adjustment. Parameters were estimated using the maximum likelihood statistical method. According to Roussel et al. [34], this method allows to have much better results. 

### 3.4. The Reliability of Measurements

Cronbach’s alpha was used as an indicator to measure the reliability of the various elements, which are supposed to help measuring a phenomenon [35]. Reliability depends on the interrelationship level (correlation, covariance) between the terms. Cortine [36] confirmed that the alpha reliability indicator gives information about the extent that each item on a scale is related to at least another element on the same scale. If the concept is really unidimensional, then all the items used to measure it will represent a single factor in the factorial analysis. In that case, the scale is characterized by a good homogeneity, as the terms measure just one dimension of the variable examined. To evaluate the dimensionality of the scales and the number of factorial axes to be kept, there are several regulations to be followed:

- The communities indicating the information quality of the starting variable, which are restored by the factor/s, obtained at the end of the analysis. In other words, the part of the variable explained by the factors used.
- The percentage of variance returned by factors; in some studies, the threshold of satisfaction is fixed a priori in terms of percentage of variance explained. It is consequently appropriate to hold the number of factors necessary to reach this threshold.
- Factorial contributions: the quality of analysis is evaluated also at the level of each variable by examining the factor loading.
- Kaiser’s criterion: If the starting point of the analysis is a correlation matrix, the classic Kaiser’s criterion consists in holding the factors whose eigenvalue is more than one. On the contrary, if it comes to a variance–covariance matrix, one can use Kaiser’s criterion to exclusively hold those factors, which represent more than 1/p% of the total variance; p is the number of affirmations on the scale. We shall also examine the KMO test to discover whether our sample is appropriate or not for the PCA, or simply to answer the question about the fact that our data are factorable or not.

With regard to the scale of sexual harassment, the variable is confirmed through the identification of three elements, which represent more than “72.956%” of the total variance explained. Sexual harassment has three main factors: risks perceived in relation to the scale of the challenge (3 items), the law of silence due to the cultural weight (6 items), and sexual harassment acts (4 items). These 13 items were minimized into 8 items, according to factor loading. The KMO index evaluated at “0.918” shows that the data about the measurement of this construct lend themselves well to the factorial analysis. As for the Bartlett test, we notice an alternative hypothesis, i.e., that the correlation matrix does not coincide with the unitary matrix (*p*-value = 0.000 < 0.05); therefore, we reject the null hypothesis. The reliability analysis shows a Cronbach’s alpha of “0.940”. It could be said that this specific measurement scale for sexual harassment is more than satisfactory. 

With regard to the trust scale in the hierarchical superior, the unidimensionality of the variable is confirmed through identification of a single element, representing over “74.731%” of the total variance explained. The KMO indicator for the data corresponding to this result is of “0.942”, thus certifying the importance of the factorial analysis on these data, especially since the *p*-value is equal to zero, which therefore excludes the null hypothesis. The reliability analysis shows a Cronbach’s alpha of “0.951”, which is excellent.

Regarding the intention to leave scale, the factorial analysis shows that the six items making up the scale by Colle et al. [33], concerning the intention to leave, are certified by identifying the two elements, which represent “81.098%” of the total variance explained. After verifying the KMO (0.765), it could be analyzed that their reliability, which displayed a Cronbach’s alpha of “0.855”, is a quite acceptable threshold. 

Finally, regarding the scale of the continuous commitment, the factorial analysis shows that the six elements adapted by Meyer and Allen [32], which measure the continuous commitment, are all related to the same factor. In fact, the unidimensionality of the continuous commitment is confirmed through the identification of the single element, which represents over “76.788%” of the total variance explained. The KMO indicator, displaying a coefficient of “0.792”, clearly proves that the datum proportional to the measurement of such a result lends itself well to the factorial analysis. The reliability analysis shows a Cronbach’s alpha of “0.939”; a very satisfactory threshold, which is very close to the result obtained by these authors. 

### 3.5. The Structural Equations Modelling Results

The validation phase of the measurement scale was performed on a sample of 305 women who work at the public hospitals. The analysis of the causal relations among the variables of the model was conducted through the methods of SEM. The choice of resorting to the structural equations derived from a series of lacks in the exploratory factorial analysis. In fact, those who use the method of the exploratory factorial analysis must grant two lacks. The first one pertains to their heuristic feature, as the factorial solution adopted is not necessarily the best one. The second one seems to be the consequence of the previous gap. Actually, the researcher is not able to compare two factorial solutions, as this method does not provide an objective means to carry out this comparison. 

The statistical software (Amos 23) was adopted to use the models of structural equations. This kind of approach makes it possible to analyze the causal connections among the variables of the theoretic model. In this respect, El Akremi [36] stated that the analysis of the covariance structure allows to “simultaneously test a great number of relations among more explanatory variables and many variables to be explained” ([37], p. 340). Moreover, Valette-Florence [38] did not hesitate to move in this direction by stating that the structural equations allow to treat numerous dependent and independent variables observed. 

The model to measure sexual harassment shows a good correspondence to the data. The ratio x^2^/df equal to “1.982” is much lower than “5”. In this regard, according to Jöreskog and Sörbom [39] the ratio is considered satisfactory when x^2^/df is lower than “5”. The RMR, “0.034” and the RMSEA, “0.057” are considered acceptable. The NFI, TLI, CFI and RFI, respectively, of “0.986, 0.989, 0.993, 0.977”, are close to “1”. The standardized RMR is equal to “0.0179”, thus being very close to “0”. The CAIC of the model is much lower than the CAIC of the saturated model, thus showing a good parsimony of the model. The Joreskög Rhô φ_JD_ is equal to “0.892”. Consequently, the scale has good reliability. 

The trust model shows a RMSEA evaluated as very good. Furthermore, the goodness of adjustment of this model is quite satisfactory. In fact, GFI, NFI, RFI and CFI show values very close to “1” [40]. The CAIC is lower than the CAIC of the saturated model, thus confirming a good thriftiness of the model. The RMR and the standardized RMR show a value very close to zero. As for the Joreskög Rhô φ_JD_, it is equal to “0.875”. Consequently, the scale has good reliability.

The model of the intention to leave shows a RMSEA of “0.073”, which is an acceptable index. Browne and Cudeck [41] confirmed it by showing that a model with a RMSEA lower than or equal to “0.08” is good. A good suitability of the model is accompanied by a good adjustment quality. GFI, AGFI, NFI, RFI, NNFI and CFI are all over “0.9” and consequently very close to “1” [39]. The square root of the mean square value of the residual values is “0.030”, i.e., close to zero. In fact, the closer this value is to “0”, the better the adjustment quality of the model. The x^2^/df ratio equal to “2.631” is very satisfactory. The CAIC is much lower than the CAIC of the saturated model, thus showing a good thriftiness of the model. The Joreskög Rhô φ_JD_ is equal to “0.911”. Therefore, the scale has good reliability. As a result, the adjustment quality of this dimension is very satisfactory. 

As for the measurement model of the continuous commitment, it shows an excellent adjustment to the data. In fact, the chi-square value is low, “4.88”, and the x^2^/df ratio, equal to “2.443”, is much lower than “5”. The RMR index, “0.015”, is excellent, as it is very close to zero. The RMSEA reports a satisfactory value of “0.069”. GFI, AGFI, NFI, NNFI, RFI and CFI are close to “1”. The CAIC of the model is much lower than the CAIC of the saturated model, thus showing a good thriftiness of the model. The Joreskög Rhô φ_JD_ is equal to “0.966”. Consequently, the scale has good reliability. The model has a quite good adjustment quality. Descriptive results of the first order confirmatory factor analysis are presented in Table 2.

It should be noted that the elements of each concept of the model significantly changed in consequence of the exploratory and confirmatory factorial analyses. In fact, during the whole purification phase of the measurement tools, the items which showed anomalies were removed. Afterward, the global quality of the adjustment of the final model was confirmed. The final model with its regression coefficients is discussed.

Average variance extracted for all the constructs was acceptable and more than 0.5 [42]. Both convergent and discriminant validities were also above 0.5, which reflect good convergent validity of the latent constructs (see Table 3). The Cronbach alpha of all the items was greater than 0.70, indicating good reliability of the scale [43].

**Figure 2:**
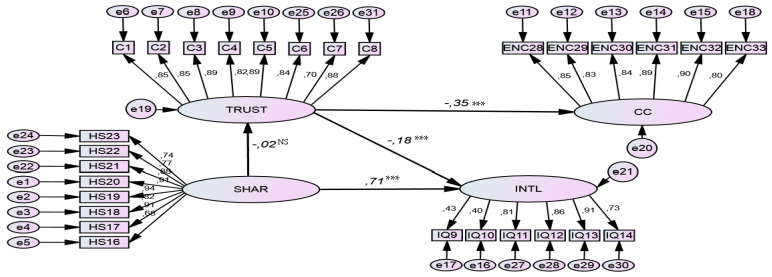
The final structural model. Trust = Trust in Superiors; SHAR = Sexual Harassment by Superiors; CC = Continuous Commitment; INTL = Intention to Leave; *** = *p* < 0.001; NS = Not Significant; Model fit: (χ2 (85, N = 305) = 330.766, *p* < 0.001, normed χ2 = 3.987, RMSEA = 0.024, SRMR = 0.136, CFI = 0.980, TLI = 0.978, NFI = 0.988, PCFI = 0.981 and PNFI = 0.970.

The model shows a RMSEA of “0.0240”, which is considered to be a satisfactory index by Didellon and Valette-Florence [40]. The good suitability of the model is accompanied by a good suitability quality, as GFI “0.981”, AGFI “0.970”, NFI “0.988”, TLI “0.978”, RFI “0.961” and CFI “0.980” are all higher than “0.96” and consequently very close to “1”, [39]. The RMR is equal to “0.017”, i.e., close to zero. The chi-square/df ratio is equal to “3.181”, an appropriate index, as recommended by Jöreskog and Sörbom [39]. The CAIC of the tested model shows an index of “160.668” lower than the CAIC of the saturated model “182.228”, thus highlighting a good parsimony of the model. In summary, this can infer that the adjustment quality of this model is satisfactory. The results of the structural model are shown in Figure 2 and Table 4.

## 4. Discussion and Implication 

According to Butler [44], trust is based on convictions about the partner or superior’s good qualities and aim. Trust is built on interpersonal relationships in perpetual motion. Therefore, trust comes from a series of subjective evaluations implied in the relational experience, upon which an individual decides to trust or not. Therefore, it describes the state of mind of a person or a group of persons, who accept the assumption that expectations, which contribute to shape the relationship with the other person, are confirmed. This corroborates the idea that to ensure the organizational commitment and individual behavior, they must be in perfect harmony with the one of the others. Nevertheless, sexual harassment by superiors toward subordinate female professionals compromises the complex moral and emotional commitments of the latter. Hence, this could be reflected in the lack of significance of the relation between sexual harassment and trust. Hence, Hypothesis 1 was not supported, and no significant influence was found between sexual harassment by superiors and subordinates’ trust in them. 

The results showed that sexual harassment by superiors is sufficiently correlated to the intention to leave. When sexual harassment increases by “1”, the intention to leave increases by “0.309”. Hence, it could be argued that the intention to leave is a behavior through which the sexually harassed woman, aware of her skills, qualifications and added value, reacts by looking for alternative jobs. This empirical observation demonstrates that sexual harassment is a real problem for young female employees. Some of them with very promising qualifications and skills can give up and finally leave their corporations and look for another job, where a healthy organizational atmosphere can let them work in safety. This causes a heavy loss of talent [45,46] and, at the same time, onerous recruiting fees for all professional circles [47].

The results showed that trust is negatively correlated to both continuous commitment and the intention to leave among young female health professionals. In fact, when trust increases by “1”, the continuous commitment and intention to leave decrease respectively by “0.319” and “0.090”. In that case, trust strengthens the perception of mutual responsibilities and associated identities [48]. Trust becomes the vector of a collaboration based on sharing the same value system [49], far from the formal or even bureaucratic aspect of the relationship [50]. 

Quite rightly, Gurviez [24] stated that in a continuous relationship, when a partner (in that case, a young female employee) trusts the other part, she will give up losing time to look for evidence that the other part is behaving properly. Therefore, if the level of trust is high, it can considerably increase the commitment level of the young female employees in the organization and consequently decrease the intention to leave, as they will feel a sense of obligation both toward their corporation and superiors. Rightly, Campoy and Neveu [51] explicitly showed that trust is the process to strengthen and improve employees’ behavior in the corporation. 

The results of current research have some implications for scholars and policymakers in the healthcare sector. For the theoretical implications, the results, interestingly, showed that trust in superiors has no mediation role between sexual harassment by superiors and young female professionals’ intention to leave nor their continuous commitment. This is because sexual harassment has no significant influence on trust in superiors. In other words, if sexual harassment by superiors takes place, young females respond directly by their intention to leave the job. Therefore, it is vital that researchers pay sufficient attention to understanding sexual harassment by superiors than in building trust in superiors. Furthermore, the current research adopted a holistic model which combines together sexual harassment by superiors, trust in superiors, continuous commitment and intention to leave, where sexual harassment is the explanatory variable. Policymakers should have a crucial role in preventing sexual harassment toward anyone, especially young female professionals, through proper legislation and regulation as well as strict implementation of these regulations. The privacy of females who report sexual harassment should be highly protected by the administration in the healthcare sector and policymakers. This will help them report any unwelcome and unethical behaviors, including sexual harassment. 

## 5. Conclusions

This research concentrated on classical hierarchical sexual harassment, i.e., the one from the immediate superior to his subordinate. The research completely avoided two further kinds of sexual harassment: horizontal sexual harassment between colleagues and what is called “counter-power harassment” in the sense given by O’Connell and Korabi [52], who discovered that 42% of their sample of women working in the academic world had been sexually harassed by men under their rank. The current research showed that sexual harassment by superiors significantly affects young female professionals’ intention to leave the job. Furthermore, the research focused only on one of the aspects of the organizational commitment [32,42,45], i.e., continuous commitment, and disregarded the other two aspects: affective and normative commitment. It is important to clarify that such a choice was purely intentional, as, highlighting this aspect, it is possible to identify more meaning that is able to meet the needs of the research question. 

The research has the following research contributions: a theoretical and a managerial one. On the theoretical level, though the literature ascertained connections between trust in superiors and intention to leave on the one hand and trust and organizational commitment on the other, there was no published research to the best of the researchers’ knowledge-without necessarily being exhaustive, which combines the three aspects at the same time, where sexual harassment is the explanatory variable. On the managerial level, our research highlighted many rules. This study revealed that sexual harassment is a very serious and even tragic problem for young female professionals. Preventing and fighting sexual harassment toward women must be taken seriously and become a challenge for corporations, which should ensure a healthy and safe environment. The ostrich policy does not solve the problem. On the contrary, cowardice allows the proliferation and encouragement of sexual harassment in the workplace. Some women employees, though deserving of successful careers, end up surrendering and dropping out of their fields of activity. This is a costly loss of talent in all professional circles. The intention to leave her company is a behavior by which a sexually harassed woman, fully aware of her skills and added value, reacts by actively looking for working alternatives.

The current research has some limitations, which could open the door for further research opportunities. Despite the fact that sexual harassment can be conducted by anyone to both males and females, the current research was limited to sexual harassment by superiors and its influence on subordinates’ trust, continuous commitment and intention to leave, particularly among young female professionals in public hospitals in three main cities of Tunisia. Hence, the results may be limited to other societies with same context and culture. Further research could examine sexual harassment in a wider context and different culture. 

## Figures and Tables

**Table 1:** The scales used in the research instrument.

Measured Variable	Authors	Number of Items
Sexual harassment	Gharbi and Sobaih [30]	13
Trust in the direct superior	Tyler and Degoey [31]	8
Continuous commitment	Meyer and Allen [32]	6
Intention to leave	Colle et al. [33]	6

**Table 2:** Descriptive results of first order confirmatory factor analysis.

Factors and Items	Loading	T-Value	M	S. D	Properties
Sexual Harassment Gharbi and Sobaih, [30] (α = 0.940)					CR = 0.946 AVE = 0.689 MSV = 0.549
I did not want to change my supervisor because my career would be over	0.68	F	3.28	1.498	
I feel compelled to continue with the same supervisor because I think that no one else would accept me	0.91	14.372	3.72	1.263	
My supervisor sees me more as a woman than a colleague or future colleague	0.81	13.001	3.82	1.230	
My supervisor understands my friendly behavior as a sign of sexual availability	0.95	15.006	3.87	1.287	
My supervisor tells me daring jokes with sexual overtones	0.90	14.260	3.72	1.368	
My supervisor is showing more and more interest in matters concerning my way of dressing	0.89	14.960	3.67	1.338	
My supervisor abuses me through phone calls	0.75	12.899	3.68	1.328	
My supervisor chooses a spatial proximity, during my supervision, deemed unnecessary	0.71	11.557	3.71	1.308	
Trust Tyler and Degoey, [31] (α = 0.951) My supervisor ……..					CR = 0.949 AVE = 0.702 MSV = 0.305
Behave honestly with me	0.86	F	3.32	1.499	
is fair to me and shows it to me	0.85	19.992	3.21	1.485	
Try her best to be fair to me	0.89	22.195	3.35	1.495	
Never cheat	0.80	17.884	3.22	1.551	
Try to be fair and upright to me	0.90	19.987	3.45	1.510	
Is sensitive to my present and future needs	0.81	18.471	3.26	1.651	
Is frank	0.71	14.832	3.32	1.449	
Communicate to me frankly the reasons for his decisions	0.87	21.194	3.31	1.455	
Continuous commitment Meyer and Allen, [32] (α = 0.855)					CR = 0.859 AVE = 0.713 MSV = 0.349
It would be very difficult for me to leave this department at this time, even if I wanted to	0.78	F	3.14	1.480	
A lot of things in my life would be disrupted if I decided to leave this department now	0.83	15.037	3.26	1.394	
At the moment, staying in this department is a problem that is as much a necessity as a desire	0.81	19.391	3.91	1.194	
I think I have too few options to consider leaving this service	0.83	23.163	3.56	1.292	
One of the negative consequences of my leaving this service would be the lack of possible alternatives	0.93	15.037	3.56	1.399	
If I hadn’t given so much of myself to this service, I might have considered working elsewhere	0.88	13.694	3.74	1.196	
The Intention to Leave Colle et al. [33] (α = 0.939)					CR = 0.937 AVE = 0.725 MSV = 0.582
I believe that I will continue to work in my current company in the future	0.78	F	3.49	1.331	
I am thinking about leaving my job in my company	0.83	15.037	3.62	1.308	
Currently, I am not actively looking for a job outside my company	0.81	19.391	3.73	1.267	
I am seriously thinking about leaving my job	0.83	23.163	3.38	1.311	
As soon as I find a more interesting job, I will leave my company	0.93	15.037	3.40	1.427	
I do not intend to leave my company in the near future	0.88	13.694	3.64	1.270	

CR = composite reliability; AVE = average variance extracted; MSV = maximum shared value.

**Table 3:** Discriminant validity.

	Sexual Harassment	Trust in Superiors	Continuous Commitment	Intention to Leave
Sexual Harassment	0.830 *			
Trust	0.566	0.838 *		
Continuous Commitment	0.423	0.565	0.713 *	
Intention To leave	0.318	0.313	0.633	0.725 *

* Diagonal values: the square root of AVE for each dimension; below diagonal values: intercorrelation between dimensions.

**Table 4:** The results of direct relationship.

Variables	Beta (β)	C.R. (T Value)	Results
SHAR 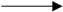 TRUST	−0.020	−0.330	Not supported
TRUST 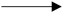 CC	−0.313 ***	−5.933	Supported
SHAR 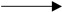 INTL	0.318 ***	6.595	Supported
TRUST 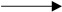 INTL	−0.080 ***	−3.571	Supported
SHAR 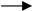 TRUST 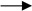 CC	Path 1: β = −0.020 Path 2: β = −0.313 ***	Path 1: t-value = −0.330 Path 2: t-value = −5.933	Not supported
SHAR 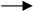 TRUST 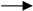 INTL	Path 1: β = −0.020 Path 2: β = −0.080 ***	Path 1: t-value = −0.330 Path 2: t-value = 3.571	Not supported

SHAR = Sexual Harassment by Superiors; Trust = Trust in Superiors; CC = Continuous Commitment; INTL = Intention to Leave; *** = *p* < 0.001.

## Data Availability

Data are available upon request from researchers who meet the eligibility criteria. Kindly contact the first author privately through the e-mail.
